# 2,3-Dibromo-3-(4-chloro­phen­yl)-1-(4-nitro­thio­phen-2-yl)propan-1-one

**DOI:** 10.1107/S1600536812034551

**Published:** 2012-08-11

**Authors:** Hoong-Kun Fun, Suhana Arshad, Shobhitha Shetty, Balakrishna Kalluraya, M. Babu

**Affiliations:** a X-ray Crystallography Unit, School of Physics, Universiti Sains Malaysia, 11800 USM, Penang, Malaysia; b Department of Studies in Chemistry, Mangalore University, Mangalagangothri 574 199, Karnataka, India

## Abstract

The title compound, C_13_H_8_Br_2_ClNO_3_S, exhibits whole-mol­ecule disorder over two orientations in a 0.805 (6):0.195 (6) ratio. The dihedral angles between the thio­phene ring [maximum deviations = 0.017 (4) and 0.033 (9) Å for the major and minor components, respectively] and the chloro-substituted phenyl ring are 32.1 (5) (major component) and 26.3 (18)° (minor component). In the crystal, C—H⋯Cl and C—H⋯O hydrogen bonds link the mol­ecules into sheets lying parallel to the *bc* plane. Aromatic π–π stacking inter­actions [centroid–centroid distance = 3.550 (7) Å] are also observed.

## Related literature

For background to nitro­thio­phene derivatives, see: Holla *et al.* (1986 ▶); Kalluraya *et al.* (1994 ▶); Kalluraya & Shetty (1997 ▶); Rai *et al.* (2008 ▶). For related structures, see: Fun *et al.* (2010 ▶, 2011 ▶). For the stability of the temperature controller used in the data collection, see: Cosier & Glazer (1986 ▶).
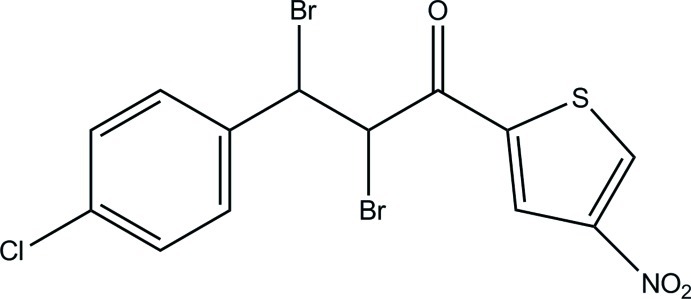


## Experimental

### 

#### Crystal data

C_13_H_8_Br_2_ClNO_3_S
*M* *_r_* = 453.53
Monoclinic, 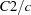

*a* = 28.5425 (17) Å
*b* = 9.5470 (5) Å
*c* = 11.4047 (7) Å
β = 103.224 (2)°
*V* = 3025.3 (3) Å^3^
*Z* = 8
Mo *K*α radiation
μ = 5.68 mm^−1^
*T* = 100 K
0.31 × 0.24 × 0.11 mm

#### Data collection

Bruker SMART APEX DUO CCD diffractometer
Absorption correction: multi-scan (*SADABS*; Bruker, 2009 ▶) *T*_min_ = 0.269, *T*_max_ = 0.564
33329 measured reflections
5031 independent reflections
4272 reflections with *I* > 2σ(*I*)
*R*_int_ = 0.057

#### Refinement

*R*[*F* ^2^ > 2σ(*F* ^2^)] = 0.041
*wR*(*F* ^2^) = 0.112
*S* = 1.11
5031 reflections
275 parameters
504 restraints
H-atom parameters constrained
Δρ_max_ = 1.33 e Å^−3^
Δρ_min_ = −0.75 e Å^−3^

### 

Data collection: *APEX2* (Bruker, 2009 ▶); cell refinement: *SAINT* (Bruker, 2009 ▶); data reduction: *SAINT*; program(s) used to solve structure: *SHELXTL* (Sheldrick, 2008 ▶); program(s) used to refine structure: *SHELXTL*; molecular graphics: *SHELXTL*; software used to prepare material for publication: *SHELXTL* and *PLATON* (Spek, 2009 ▶).

## Supplementary Material

Crystal structure: contains datablock(s) global, I. DOI: 10.1107/S1600536812034551/hb6917sup1.cif

Structure factors: contains datablock(s) I. DOI: 10.1107/S1600536812034551/hb6917Isup2.hkl

Supplementary material file. DOI: 10.1107/S1600536812034551/hb6917Isup3.cml

Additional supplementary materials:  crystallographic information; 3D view; checkCIF report

## Figures and Tables

**Table 1:** Hydrogen-bond geometry (Å, °)

*D*—H⋯*A*	*D*—H	H⋯*A*	*D*⋯*A*	*D*—H⋯*A*
C7—H7*BA*⋯Cl1^i^	1.00	2.82	3.441 (4)	121
C11—H11*B*⋯O1^ii^	0.95	2.49	3.435 (6)	175

Symmetry codes: (i) 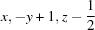; (ii) 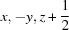.

**Fig. 1.**
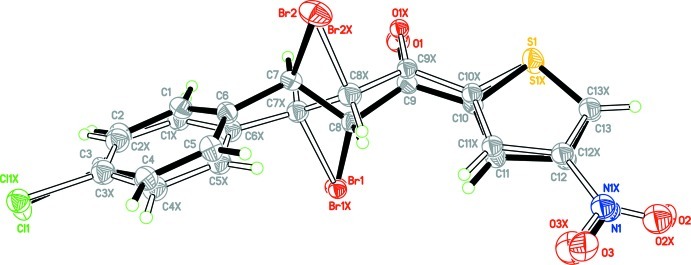
The molecular structure of the title compound, showing 50% probability displacement ellipsoids. Both disorder components are shown.

**Fig. 2.**
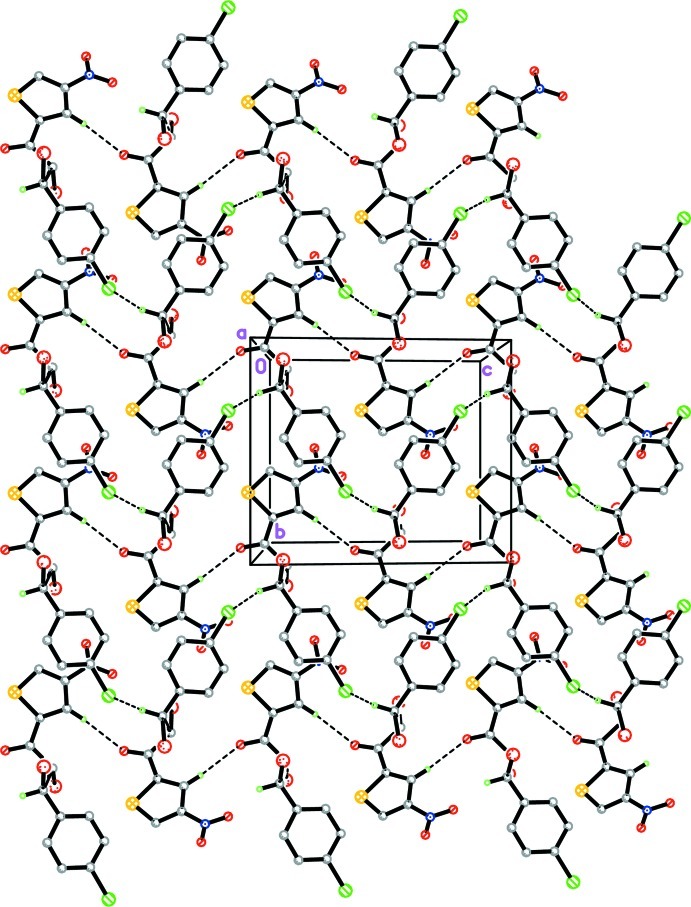
The crystal packing of the title compound. Dashed lines represent the hydrogen bonds. Only major disorder component is shown.

## supplementary crystallographic information

### Comment

Nitrothiophene and its derivatives possess a wide variety of pharmacological activity. The presence of a nitro group at the 4-position of the molecule conferred antibacterial activity (Holla *et al.*, 1986). A large number of nitrothiophene derivatives are reported to exhibit a variety of biological activity such as antibacterial, antifungal *etc*. (Kalluraya *et al.*, 1994; Kalluraya & Shetty, 1997). Dibromopropanones were obtained by the bromination of 1-aryl-3-(4-nitro-2-thienyl)-2-propen-1-ones. Acid-catalysed condensation of acetophenones with 4-nitrothiophenediacetate in acetic acid yielded the required 1-aryl-3-(4-nitro-2-thienyl)-2-propen-1-ones known as chalcones (Rai *et al.*, 2008).

The molecular structure is shown in Fig. 1. Bond lengths and angles are within normal ranges and comparable to the related structures (Fun *et al.*, 2010; Fun *et al.*, 2011). The whole molecule of the title compound is disordered over two positions with a refined site-occupancies ratio of 0.805 (6): 0.195 (6). For the major disorder component, the thiophene ring (S1/C10–C13) is approximately planar with maximum deviation of 0.017 (4) Å at atom C10 and forms a dihedral angle of 32.1 (5)° with the chloro-substituted phenyl ring (C1–C6). Meanwhile, for the minor disorder component, the approximately planar thiophene ring [S1X/C10X–C13X, with maximum deviation of 0.033 (9) Å at atom S1X] makes a dihedral angle of 26.3 (18)° with the chloro-substituted phenyl ring (C1X–C6X).

In the crystal (Fig. 2), C7—H7BA···Cl1 and C11—H11A···O1 hydrogen bonds (Table 1) link the molecules into a two-dimensional network parallel to the *bc*-plane. π–π interaction of *Cg*1···*Cg*1 = 3.550 (7) Å (symmetry code: 1/2 - *x*, -1/2 - *y*,-*z*) consolidate the crystal structure [*Cg*1 is the centroid of the major component of the thiophene ring (S1/C10–C13)].

### Experimental

3-(4-Chlorophenyl)-1-(4-nitrothiophen-2-yl)prop-2-en-1-one (0.01 mol) was dissolved in glacial acetic acid (25 ml) by gentle warming. A solution of bromine in glacial acetic acid (30% *w*/*v*) was added to it with constant stirring until the yellow color of the bromine persisted. The reaction mixture was kept aside at room temperature for overnight. Crystals of dibromopropanone that separated out were collected by filtration and washed with petroleum ether and dried. They were then recrystallized from glacial acetic acid. Colourless blocks were obtained from 1:2 mixtures of DMF and ethanol solution by slow evaporation.

### Refinement

The title compound is disordered over two sets of positions with a refined site-occupancies ratio of 0.805 (6): 0.195 (6). The minor disorder component was refined isotropically. All disordered atoms were subjected to similarity restraints (SAME) except for atoms Br1X, Br2X, C7X and C8X. The similar-ADP restraint (SIMU) was applied to all atoms in the molecule. A FLAT restraint was also used to the minor component of the chloro-phenyl ring (Cl1X/C1X–C6X). All H atoms were positioned geometrically [C–H = 0.95 and 1.00 Å] and refined using a riding model with *U*_iso_(H) = 1.2 *U*_eq_(C).

### Crystal data

**Table:**

C_13_H_8_Br_2_ClNO_3_S	*F*(000) = 1760
*M**_r_* = 453.53	*D*_x_ = 1.991 Mg m^−^^3^
Monoclinic, *C*2/*c*	Mo *K*α radiation, λ = 0.71073 Å
Hall symbol: -C 2yc	Cell parameters from 9951 reflections
*a* = 28.5425 (17) Å	θ = 2.8–31.4°
*b* = 9.5470 (5) Å	µ = 5.68 mm^−^^1^
*c* = 11.4047 (7) Å	*T* = 100 K
β = 103.224 (2)°	Block, colourless
*V* = 3025.3 (3) Å^3^	0.31 × 0.24 × 0.11 mm
*Z* = 8	

### Data collection

**Table:**

Bruker SMART APEX DUO CCD diffractometer	5031 independent reflections
Radiation source: fine-focus sealed tube	4272 reflections with *I* > 2σ(*I*)
Graphite monochromator	*R*_int_ = 0.057
φ and ω scans	θ_max_ = 31.6°, θ_min_ = 1.5°
Absorption correction: multi-scan (*SADABS*; Bruker, 2009)	*h* = −41→41
*T*_min_ = 0.269, *T*_max_ = 0.564	*k* = −14→14
33329 measured reflections	*l* = −16→16

### Refinement

**Table:**

Refinement on *F*^2^	Primary atom site location: structure-invariant direct methods
Least-squares matrix: full	Secondary atom site location: difference Fourier map
*R*[*F*^2^ > 2σ(*F*^2^)] = 0.041	Hydrogen site location: inferred from neighbouring sites
*wR*(*F*^2^) = 0.112	H-atom parameters constrained
*S* = 1.11	*w* = 1/[σ^2^(*F*_o_^2^) + (0.0628*P*)^2^ + 5.4597*P*] where *P* = (*F*_o_^2^ + 2*F*_c_^2^)/3
5031 reflections	(Δ/σ)_max_ = 0.001
275 parameters	Δρ_max_ = 1.33 e Å^−^^3^
504 restraints	Δρ_min_ = −0.75 e Å^−^^3^

### Special details

**Table:**

Experimental. The crystal was placed in the cold stream of an Oxford Cryosystems Cobra open-flow nitrogen cryostat (Cosier & Glazer, 1986) operating at 100.0 (1) K.
Geometry. All e.s.d.'s (except the e.s.d. in the dihedral angle between two l.s. planes) are estimated using the full covariance matrix. The cell e.s.d.'s are taken into account individually in the estimation of e.s.d.'s in distances, angles and torsion angles; correlations between e.s.d.'s in cell parameters are only used when they are defined by crystal symmetry. An approximate (isotropic) treatment of cell e.s.d.'s is used for estimating e.s.d.'s involving l.s. planes.
Refinement. Refinement of *F*^2^ against ALL reflections. The weighted *R*-factor *wR* and goodness of fit *S* are based on *F*^2^, conventional *R*-factors *R* are based on *F*, with *F* set to zero for negative *F*^2^. The threshold expression of *F*^2^ > σ(*F*^2^) is used only for calculating *R*-factors(gt) *etc*. and is not relevant to the choice of reflections for refinement. *R*-factors based on *F*^2^ are statistically about twice as large as those based on *F*, and *R*- factors based on ALL data will be even larger.

### Fractional atomic coordinates and isotropic or equivalent isotropic displacement parameters (Å^2^)

**Table:**

	*x*	*y*	*z*	*U*_iso_*/*U*_eq_	Occ. (<1)
Br1	0.30443 (7)	0.1934 (2)	0.09470 (18)	0.0220 (2)	0.805 (6)
Br2	0.45859 (4)	0.04291 (14)	0.08975 (14)	0.0328 (3)	0.805 (6)
Cl1	0.46000 (9)	0.7011 (2)	0.3575 (2)	0.0325 (4)	0.805 (6)
S1	0.31346 (12)	−0.2888 (4)	−0.0725 (3)	0.0278 (8)	0.805 (6)
O1	0.35091 (17)	−0.0087 (5)	−0.1008 (4)	0.0250 (8)	0.805 (6)
O2	0.2644 (3)	−0.5160 (6)	0.2173 (6)	0.0280 (10)	0.805 (6)
O3	0.3097 (3)	−0.3704 (7)	0.3399 (4)	0.0359 (11)	0.805 (6)
N1	0.29043 (19)	−0.4130 (6)	0.2375 (4)	0.0219 (9)	0.805 (6)
C9	0.34861 (14)	−0.0298 (3)	0.0020 (3)	0.0196 (7)	0.805 (6)
C10	0.33008 (15)	−0.1624 (4)	0.0378 (3)	0.0186 (8)	0.805 (6)
C11	0.32325 (14)	−0.2035 (4)	0.1496 (4)	0.0191 (8)	0.805 (6)
H11B	0.3313	−0.1501	0.2217	0.023*	0.805 (6)
C12	0.3022 (2)	−0.3397 (6)	0.1375 (4)	0.0199 (9)	0.805 (6)
C13	0.2942 (8)	−0.3972 (12)	0.0240 (6)	0.0211 (14)	0.805 (6)
H13B	0.2797	−0.4860	0.0026	0.025*	0.805 (6)
C1	0.40955 (16)	0.4341 (4)	0.0905 (4)	0.0228 (9)	0.805 (6)
H1BA	0.3958	0.4389	0.0064	0.027*	0.805 (6)
C2	0.4226 (2)	0.5568 (6)	0.1542 (5)	0.0246 (9)	0.805 (6)
H2A	0.4178	0.6452	0.1149	0.029*	0.805 (6)
C3	0.4427 (4)	0.5479 (5)	0.2764 (5)	0.0226 (9)	0.805 (6)
C4	0.44978 (18)	0.4192 (5)	0.3367 (4)	0.0242 (9)	0.805 (6)
H4BA	0.4636	0.4150	0.4207	0.029*	0.805 (6)
C5	0.43608 (16)	0.2978 (4)	0.2707 (4)	0.0239 (7)	0.805 (6)
H5A	0.4403	0.2095	0.3101	0.029*	0.805 (6)
C6	0.41604 (14)	0.3040 (3)	0.1464 (3)	0.0202 (6)	0.805 (6)
C7	0.40208 (11)	0.1763 (3)	0.0704 (3)	0.0195 (6)	0.805 (6)
H7BA	0.3919	0.2052	−0.0159	0.023*	0.805 (6)
C8	0.36384 (11)	0.0820 (3)	0.1003 (3)	0.0186 (6)	0.805 (6)
H8BA	0.3754	0.0376	0.1812	0.022*	0.805 (6)
Br1X	0.3073 (3)	0.1867 (8)	0.1063 (7)	0.0159 (8)*	0.195 (6)
Br2X	0.45697 (15)	0.0543 (4)	0.0743 (4)	0.0118 (6)*	0.195 (6)
Cl1X	0.4684 (3)	0.6876 (9)	0.3684 (9)	0.0225 (15)*	0.195 (6)
S1X	0.3131 (3)	−0.2879 (11)	−0.0739 (10)	0.0085 (17)*	0.195 (6)
O1X	0.3601 (6)	−0.023 (2)	−0.1041 (15)	0.016 (3)*	0.195 (6)
O2X	0.2680 (13)	−0.499 (3)	0.225 (3)	0.035 (5)*	0.195 (6)
O3X	0.3116 (13)	−0.348 (3)	0.343 (2)	0.041 (5)*	0.195 (6)
N1X	0.2988 (10)	−0.408 (3)	0.2442 (19)	0.023 (3)*	0.195 (6)
C9X	0.3611 (6)	−0.0443 (15)	−0.0001 (15)	0.018 (2)*	0.195 (6)
C10X	0.3400 (7)	−0.1710 (17)	0.0376 (15)	0.018 (3)*	0.195 (6)
C11X	0.3338 (7)	−0.208 (2)	0.1501 (16)	0.019 (3)*	0.195 (6)
H11A	0.3440	−0.1547	0.2218	0.023*	0.195 (6)
C12X	0.3095 (12)	−0.338 (3)	0.1411 (19)	0.020 (2)*	0.195 (6)
C13X	0.298 (3)	−0.396 (5)	0.028 (2)	0.021 (3)*	0.195 (6)
H13A	0.2827	−0.4853	0.0091	0.026*	0.195 (6)
C1X	0.4024 (8)	0.4497 (18)	0.0976 (19)	0.022 (3)*	0.195 (6)
H1A	0.3894	0.4634	0.0141	0.027*	0.195 (6)
C2X	0.4233 (8)	0.561 (2)	0.168 (2)	0.025 (3)*	0.195 (6)
H2BA	0.4233	0.6515	0.1337	0.030*	0.195 (6)
C3X	0.4443 (17)	0.539 (2)	0.288 (2)	0.024 (3)*	0.195 (6)
C4X	0.4420 (9)	0.410 (2)	0.343 (2)	0.024 (3)*	0.195 (6)
H4A	0.4535	0.3983	0.4277	0.029*	0.195 (6)
C5X	0.4221 (6)	0.2990 (17)	0.2698 (14)	0.022 (2)*	0.195 (6)
H5BA	0.4232	0.2075	0.3030	0.027*	0.195 (6)
C6X	0.4003 (6)	0.3189 (15)	0.1474 (14)	0.023 (2)*	0.195 (6)
C7X	0.3736 (5)	0.1993 (13)	0.0709 (13)	0.022 (2)*	0.195 (6)
H7A	0.3712	0.2173	−0.0167	0.026*	0.195 (6)
C8X	0.3891 (5)	0.0534 (14)	0.1024 (14)	0.024 (2)*	0.195 (6)
H8A	0.3871	0.0244	0.1854	0.029*	0.195 (6)

### Atomic displacement parameters (Å^2^)

**Table:**

	*U*^11^	*U*^22^	*U*^33^	*U*^12^	*U*^13^	*U*^23^
Br1	0.0208 (4)	0.0242 (4)	0.0211 (5)	0.0032 (2)	0.0050 (3)	−0.0006 (3)
Br2	0.0255 (2)	0.0390 (4)	0.0325 (6)	0.0105 (2)	0.0038 (3)	−0.0058 (3)
Cl1	0.0392 (10)	0.0234 (6)	0.0339 (8)	−0.0051 (6)	0.0061 (7)	−0.0101 (5)
S1	0.0432 (11)	0.0217 (7)	0.0185 (7)	−0.0039 (3)	0.0069 (4)	−0.0021 (3)
O1	0.030 (2)	0.0236 (15)	0.0216 (14)	0.0009 (14)	0.0072 (13)	0.0021 (10)
O2	0.040 (2)	0.0171 (17)	0.031 (2)	−0.0044 (12)	0.0148 (15)	0.0032 (14)
O3	0.061 (2)	0.035 (2)	0.0115 (13)	−0.0088 (19)	0.0079 (12)	0.0039 (12)
N1	0.027 (2)	0.0211 (14)	0.0198 (14)	0.0022 (16)	0.0091 (14)	0.0028 (11)
C9	0.0198 (15)	0.0191 (13)	0.0197 (14)	0.0005 (11)	0.0043 (12)	−0.0030 (10)
C10	0.0210 (18)	0.0182 (13)	0.0167 (14)	0.0012 (12)	0.0042 (13)	−0.0014 (10)
C11	0.0191 (18)	0.0176 (12)	0.0204 (15)	0.0010 (13)	0.0040 (13)	−0.0006 (10)
C12	0.024 (3)	0.0178 (12)	0.0185 (14)	0.0002 (14)	0.0063 (14)	0.0004 (10)
C13	0.028 (4)	0.0171 (13)	0.0187 (15)	0.0002 (14)	0.0058 (17)	−0.0003 (11)
C1	0.0227 (18)	0.0226 (15)	0.0231 (16)	0.0032 (12)	0.0048 (13)	−0.0021 (12)
C2	0.0262 (15)	0.0213 (14)	0.026 (2)	0.0008 (10)	0.0051 (14)	−0.0017 (13)
C3	0.0257 (16)	0.0191 (14)	0.0224 (19)	−0.0040 (14)	0.0038 (16)	−0.0066 (13)
C4	0.025 (2)	0.0268 (15)	0.0207 (16)	−0.0038 (13)	0.0048 (14)	−0.0034 (12)
C5	0.0247 (17)	0.0219 (13)	0.0238 (16)	−0.0002 (13)	0.0030 (14)	−0.0018 (11)
C6	0.0194 (14)	0.0200 (13)	0.0208 (14)	−0.0001 (10)	0.0041 (12)	−0.0020 (10)
C7	0.0209 (13)	0.0196 (12)	0.0182 (14)	0.0017 (9)	0.0052 (10)	−0.0009 (10)
C8	0.0224 (13)	0.0158 (11)	0.0173 (13)	−0.0007 (10)	0.0042 (10)	0.0005 (10)

### Geometric parameters (Å, º)

**Table:**

Br1—C8	1.990 (4)	Br1X—C7X	2.029 (16)
Br2—C7	2.027 (3)	Br2X—C8X	2.034 (16)
Cl1—C3	1.741 (4)	Cl1X—C3X	1.742 (14)
S1—C13	1.691 (5)	S1X—C13X	1.687 (15)
S1—C10	1.730 (4)	S1X—C10X	1.733 (14)
O1—C9	1.206 (5)	O1X—C9X	1.198 (15)
O2—N1	1.223 (5)	O2X—N1X	1.216 (15)
O3—N1	1.239 (5)	O3X—N1X	1.240 (16)
N1—C12	1.441 (4)	N1X—C12X	1.444 (14)
C9—C10	1.466 (4)	C9X—C10X	1.459 (14)
C9—C8	1.537 (4)	C9X—C8X	1.56 (2)
C10—C11	1.390 (5)	C10X—C11X	1.381 (15)
C11—C12	1.426 (5)	C11X—C12X	1.419 (15)
C11—H11B	0.9500	C11X—H11A	0.9500
C12—C13	1.377 (5)	C12X—C13X	1.376 (15)
C13—H13B	0.9500	C13X—H13A	0.9500
C1—C2	1.384 (5)	C1X—C6X	1.378 (15)
C1—C6	1.389 (5)	C1X—C2X	1.379 (15)
C1—H1BA	0.9500	C1X—H1A	0.9500
C2—C3	1.383 (5)	C2X—C3X	1.378 (15)
C2—H2A	0.9500	C2X—H2BA	0.9500
C3—C4	1.400 (5)	C3X—C4X	1.395 (15)
C4—C5	1.389 (5)	C4X—C5X	1.390 (15)
C4—H4BA	0.9500	C4X—H4A	0.9500
C5—C6	1.403 (5)	C5X—C6X	1.404 (15)
C5—H5A	0.9500	C5X—H5BA	0.9500
C6—C7	1.496 (4)	C6X—C7X	1.531 (19)
C7—C8	1.513 (4)	C7X—C8X	1.480 (19)
C7—H7BA	1.0000	C7X—H7A	1.0000
C8—H8BA	1.0000	C8X—H8A	1.0000
			
C13—S1—C10	91.7 (3)	C13X—S1X—C10X	91.9 (9)
O2—N1—O3	124.1 (5)	O2X—N1X—O3X	123 (2)
O2—N1—C12	118.8 (4)	O2X—N1X—C12X	117 (2)
O3—N1—C12	116.9 (4)	O3X—N1X—C12X	116.6 (19)
O1—C9—C10	121.2 (4)	O1X—C9X—C10X	121.2 (16)
O1—C9—C8	121.6 (3)	O1X—C9X—C8X	121.9 (15)
C10—C9—C8	117.2 (3)	C10X—C9X—C8X	116.7 (13)
C11—C10—C9	129.4 (3)	C11X—C10X—C9X	129.4 (14)
C11—C10—S1	113.5 (3)	C11X—C10X—S1X	112.8 (11)
C9—C10—S1	117.1 (3)	C9X—C10X—S1X	117.4 (11)
C10—C11—C12	108.2 (3)	C10X—C11X—C12X	108.8 (13)
C10—C11—H11B	125.9	C10X—C11X—H11A	125.6
C12—C11—H11B	125.9	C12X—C11X—H11A	125.6
C13—C12—C11	115.5 (4)	C13X—C12X—C11X	115.6 (14)
C13—C12—N1	122.2 (4)	C13X—C12X—N1X	121.8 (15)
C11—C12—N1	122.4 (4)	C11X—C12X—N1X	122.5 (16)
C12—C13—S1	111.1 (3)	C12X—C13X—S1X	110.5 (13)
C12—C13—H13B	124.5	C12X—C13X—H13A	124.7
S1—C13—H13B	124.5	S1X—C13X—H13A	124.7
C2—C1—C6	121.6 (4)	C6X—C1X—C2X	120.7 (17)
C2—C1—H1BA	119.2	C6X—C1X—H1A	119.6
C6—C1—H1BA	119.2	C2X—C1X—H1A	119.6
C3—C2—C1	118.4 (4)	C3X—C2X—C1X	119.6 (18)
C3—C2—H2A	120.8	C3X—C2X—H2BA	120.2
C1—C2—H2A	120.8	C1X—C2X—H2BA	120.2
C2—C3—C4	122.0 (4)	C2X—C3X—C4X	121.9 (15)
C2—C3—Cl1	119.0 (4)	C2X—C3X—Cl1X	115.6 (14)
C4—C3—Cl1	119.0 (4)	C4X—C3X—Cl1X	122.3 (15)
C5—C4—C3	118.3 (4)	C5X—C4X—C3X	117.1 (16)
C5—C4—H4BA	120.8	C5X—C4X—H4A	121.5
C3—C4—H4BA	120.8	C3X—C4X—H4A	121.5
C4—C5—C6	120.8 (4)	C4X—C5X—C6X	121.6 (15)
C4—C5—H5A	119.6	C4X—C5X—H5BA	119.2
C6—C5—H5A	119.6	C6X—C5X—H5BA	119.2
C1—C6—C5	118.9 (3)	C1X—C6X—C5X	118.7 (14)
C1—C6—C7	118.2 (3)	C1X—C6X—C7X	120.3 (14)
C5—C6—C7	122.9 (3)	C5X—C6X—C7X	121.0 (13)
C6—C7—C8	117.7 (3)	C8X—C7X—C6X	118.8 (12)
C6—C7—Br2	110.4 (2)	C8X—C7X—Br1X	97.8 (10)
C8—C7—Br2	101.46 (19)	C6X—C7X—Br1X	107.2 (10)
C6—C7—H7BA	108.9	C8X—C7X—H7A	110.7
C8—C7—H7BA	108.9	C6X—C7X—H7A	110.7
Br2—C7—H7BA	108.9	Br1X—C7X—H7A	110.7
C7—C8—C9	110.3 (3)	C7X—C8X—C9X	107.9 (12)
C7—C8—Br1	109.2 (2)	C7X—C8X—Br2X	101.5 (9)
C9—C8—Br1	104.5 (2)	C9X—C8X—Br2X	103.1 (10)
C7—C8—H8BA	110.9	C7X—C8X—H8A	114.3
C9—C8—H8BA	110.9	C9X—C8X—H8A	114.3
Br1—C8—H8BA	110.9	Br2X—C8X—H8A	114.3
			
O1—C9—C10—C11	−177.6 (4)	O1X—C9X—C10X—C11X	−173 (2)
C8—C9—C10—C11	1.1 (6)	C8X—C9X—C10X—C11X	12 (3)
O1—C9—C10—S1	1.9 (6)	O1X—C9X—C10X—S1X	−1 (3)
C8—C9—C10—S1	−179.4 (3)	C8X—C9X—C10X—S1X	−174.9 (13)
C13—S1—C10—C11	2.8 (9)	C13X—S1X—C10X—C11X	−5 (4)
C13—S1—C10—C9	−176.8 (9)	C13X—S1X—C10X—C9X	−179 (4)
C9—C10—C11—C12	177.2 (4)	C9X—C10X—C11X—C12X	177 (2)
S1—C10—C11—C12	−2.3 (5)	S1X—C10X—C11X—C12X	4 (3)
C10—C11—C12—C13	0.5 (12)	C10X—C11X—C12X—C13X	−1 (6)
C10—C11—C12—N1	179.9 (5)	C10X—C11X—C12X—N1X	178 (3)
O2—N1—C12—C13	−14.5 (14)	O2X—N1X—C12X—C13X	−20 (7)
O3—N1—C12—C13	162.0 (13)	O3X—N1X—C12X—C13X	179 (6)
O2—N1—C12—C11	166.2 (7)	O2X—N1X—C12X—C11X	162 (4)
O3—N1—C12—C11	−17.3 (9)	O3X—N1X—C12X—C11X	1 (5)
C11—C12—C13—S1	1.5 (17)	C11X—C12X—C13X—S1X	−3 (8)
N1—C12—C13—S1	−177.9 (8)	N1X—C12X—C13X—S1X	178 (4)
C10—S1—C13—C12	−2.4 (14)	C10X—S1X—C13X—C12X	5 (6)
C6—C1—C2—C3	0.3 (8)	C6X—C1X—C2X—C3X	3 (3)
C1—C2—C3—C4	−0.6 (13)	C1X—C2X—C3X—C4X	−5 (5)
C1—C2—C3—Cl1	179.2 (5)	C1X—C2X—C3X—Cl1X	−178.7 (17)
C2—C3—C4—C5	0.2 (13)	C2X—C3X—C4X—C5X	7 (6)
Cl1—C3—C4—C5	−179.6 (5)	Cl1X—C3X—C4X—C5X	−180 (3)
C3—C4—C5—C6	0.5 (8)	C3X—C4X—C5X—C6X	−7 (4)
C2—C1—C6—C5	0.3 (6)	C2X—C1X—C6X—C5X	−3 (2)
C2—C1—C6—C7	−178.6 (4)	C2X—C1X—C6X—C7X	175.2 (11)
C4—C5—C6—C1	−0.8 (6)	C4X—C5X—C6X—C1X	5 (3)
C4—C5—C6—C7	178.1 (4)	C4X—C5X—C6X—C7X	−173.1 (18)
C1—C6—C7—C8	−119.9 (4)	C1X—C6X—C7X—C8X	152.4 (17)
C5—C6—C7—C8	61.3 (5)	C5X—C6X—C7X—C8X	−29 (2)
C1—C6—C7—Br2	124.3 (3)	C1X—C6X—C7X—Br1X	−98.1 (18)
C5—C6—C7—Br2	−54.5 (4)	C5X—C6X—C7X—Br1X	80.3 (17)
C6—C7—C8—C9	171.2 (3)	C6X—C7X—C8X—C9X	−171.9 (13)
Br2—C7—C8—C9	−68.2 (3)	Br1X—C7X—C8X—C9X	73.4 (12)
C6—C7—C8—Br1	56.9 (3)	C6X—C7X—C8X—Br2X	−63.9 (15)
Br2—C7—C8—Br1	177.45 (15)	Br1X—C7X—C8X—Br2X	−178.6 (6)
O1—C9—C8—C7	−28.4 (5)	O1X—C9X—C8X—C7X	51 (2)
C10—C9—C8—C7	153.0 (3)	C10X—C9X—C8X—C7X	−134.3 (16)
O1—C9—C8—Br1	88.9 (4)	O1X—C9X—C8X—Br2X	−55.5 (18)
C10—C9—C8—Br1	−89.8 (3)	C10X—C9X—C8X—Br2X	118.8 (14)

### Hydrogen-bond geometry (Å, º)

**Table:**

*D*—H···*A*	*D*—H	H···*A*	*D*···*A*	*D*—H···*A*
C7—H7*BA*···Cl1^i^	1.00	2.82	3.441 (4)	121
C11—H11*B*···O1^ii^	0.95	2.49	3.435 (6)	175

Symmetry codes: (i) *x*, −*y*+1, *z*−1/2; (ii) *x*, −*y*, *z*+1/2.
